# Working While Ill Is Not Always Bad—Positive Effects of Presenteeism

**DOI:** 10.3389/fpsyg.2020.620918

**Published:** 2021-01-22

**Authors:** Daniela Lohaus, Wolfgang Habermann, Isam El Kertoubi, Florian Röser

**Affiliations:** ^1^Business Psychology Institute, Social Sciences Faculty, Darmstadt University of Applied Sciences, Darmstadt, Germany; ^2^H & L Karriereberatung, Lautertal, Germany

**Keywords:** presenteeism, health, productivity, positive effects, health belief model

## Abstract

Presenteeism—going to work while ill—is a widespread phenomenon worldwide. Previous research has concentrated mainly on its negative effects. This study investigates the positive consequences of presenteeism derived from a comprehensive content model of presenteeism that was developed on the basis of negative effects. In a quantitative online-survey employees (*N* = 181) rated the degree of experienced or potential positive effects depending on whether they had worked while ill (75%) or not (25%) during the previous year. Results revealed that all postulated positive effects described in the content model were relevant. Most positive effects were rated significantly higher by participants who had shown presenteeism in comparison to those who had not. The positive effects significantly predicted presenteeism propensity (adjusted *R*^2^ = 0.20) for participants having shown presenteeism. In addition, an overall rating of positive effects was significantly related to presenteeism, however, to a lesser degree. Overall, the results demonstrate the applicability of the content model to positive effects of presenteeism. They point to the need for further investigation of them and their consideration for the management of presenteeism.

## Introduction

Presenteeism, which is often seen as the opposite of absenteeism, the absence from work due to sickness, has a comparatively short research history (Johns, 2010). Although different definitions are in use, “consensus is now emerging that presenteeism describes attending work when one is unwell” (Karanika-Murray and Cooper, 2018, p. 11). In view of the unprecedented emergence of the highly infectious virus COVID-19, it has to be said that the understanding of presenteeism does not cover such illnesses. Presenteeism is a worldwide-observed behavior with prevalence rates over a year varying between 30% and over 90% of questioned employees (Lohaus and Habermann, 2018). Depending on the definition of presenteeism, costs associated with it presumably exceed those caused by absenteeism (e.g., Collins et al., 2005; Halbesleben et al., 2014). Though the majority of studies are cross-sectional (Miraglia and Johns, 2016), there are also longitudinal studies. They mainly focus on the negative consequences for the organization, such as productivity loss (e.g., Collins et al., 2005; Goetzel et al., 2009; Zhou et al., 2016; Schmid et al., 2017; Strömberg et al., 2017; Vänni et al., 2017), and for the individual, e.g., impaired health conditions in the future (e.g., Gustafsson and Marklund, 2011; Lu et al., 2013; Skagen and Collins, 2016).

Only recently, researchers increasingly argue that the sole focus on negative effects of presenteeism lacks consideration of positive consequences and they point to positive effects (Demerouti et al., 2009; Steinke and Badura, 2011; Garrow, 2016; Giæver et al., 2016; Karanika-Murray and Cooper, 2018; Miraglia and Johns, 2018; Karanika-Murray and Biron, 2020; Ruhle et al., 2020). For example, researchers investigating reasons for presenteeism imply positive effects in their answer options (e.g., Lu et al., 2013; Johansen et al., 2014; Krane et al., 2014; Gerich, 2020) and stress the potential salutogenic effects of presenteeism (Gerich, 2020). However, empirical evidence is still scarce and unsystematic (Karanika-Murray and Biron, 2020). Thus, the aim of the current study was to investigate potential and experienced positive consequences of presenteeism in a systematic way by using a category system derived from the to-date most comprehensive model of associated factors (Lohaus and Habermann, 2019, 2020).

### Theoretical Approaches and Evidence of Positive Effects of Presenteeism

The assumption of positive effects of presenteeism is plausible, since work in general can be beneficial for health and well-being, can convey a sense of significance (Rosso et al., 2010; Miraglia and Johns, 2018), and can help to fulfill psychological needs (Van den Broeck et al., 2016). Measures of gradual reintegration into their daily work routine for long-term sick employees while they are still convalescent correspond to these findings (e.g., Howard et al., 2009). Scandinavian researchers have argued for some years that organizational supportive measures to adjust the work to sickness-induced requirements would allow employees to work in spite of illness (e.g., Thun et al., 2013; Rostad et al., 2015; Thun, 2017).

The health belief model (Janz and Becker, 1984) belongs to the most frequently cited and researched models of health-related behavior (Harrison et al., 1992). In accordance with expectancy-value-models, it explains rational behavior under conditions of uncertainty. It describes health-related behavior as a result of a subjective evaluation process and implies the occurrence of presenteeism. According to it, affected employees assess their health threats or risks by evaluating their own vulnerability and the severity of their illness. Subjectively experienced health status and presenteeism show a close statistical relationship (Gerich, 2016; Miraglia and Johns, 2016). Thus, it is an obvious assumption that individuals, who view themselves as less vulnerable and more robust in general and who experience an actual health impairment as less serious, tend to show more presenteeism than people who assess those aspects as less favorable. Further, according to the health belief model, individuals take into account the barriers or costs for their behavior (e.g., “If I work in spite of sickness, I might suffer from increased pain.”) and its benefits (e.g., “If I work in spite of sickness, I contribute to the achievement of company goals.”). Hence, employees presumably show presenteeism, if they conclude that in face of a reasonable threat for their health, the expected positive effects outweigh the expected negative consequences.

Karanika-Murray and Biron (2020) postulate a framework that conceptualizes presenteeism as a behavior of employees intended to balance performance requirements with health impairments that require recovery: “Thus, we define presenteeism as goal-directed and purposeful attendance behavior aimed at facilitating adaptation to work in the face of compromised health.” (p. 245). They take the aforementioned assumptions one step further by supposing that employees follow the goals of fulfilling work requirements and at the same time strive for benefits for their physical and mental health when deciding for presenteeism. With regard to positive consequences of presenteeism, they distinguish two kinds of presenteeism. If employees manage to work (nearly) to their full capacity and at the same time recover at least to a certain degree from their health impairments, they speak of functional presenteeism. A precondition for functional presenteeism is a supportive work situation that offers sufficient resources. In contrast to functional presenteeism, they describe cases in which employees perform on a considerably lower level than usual; however, their health benefits from attending work. They label this behavior therapeutic presenteeism, which also profits from resources in the work environment. Positive effects on the health of employees might arise from a positive team atmosphere and support from colleagues. Working at all in the face of illness could be beneficial for the employees' sense of responsibility, their self-esteem and self-efficacy. Employees attending work while ill and not being very productive might still receive positive feedback for their conscientiousness, their (however minor) contribution, and their team spirit. In addition, they spare their team members having to replace them (Dew et al., 2005; Caverley et al., 2007). Further, presenteeism might have the effect of experiencing joy and satisfaction from performing one's duty or helping one's clients or patients (Giæver et al., 2016). It might also divert from disease symptoms (Miraglia and Johns, 2018).

There are further considerations with regard to positive consequences of working through illness for the employer and the society as a whole. Researchers highlight the fact that most employees that show presenteeism have at least any productivity in comparison to nil when being absent (Vingård et al., 2004; Johns, 2010). Thus, at least in the short run, they contribute to economic growth and do not burden social welfare and security systems (Lohaus and Habermann, 2019).

### Frame of Reference for the Systematic Investigation of Positive Effects of Presenteeism

In order to investigate potential and actual positive consequences of presenteeism behavior, we refer to an established and to-date most comprehensive content model describing the emergence of presenteeism (Lohaus and Habermann, 2019). It takes into consideration inter alia the results of the meta-analysis by Miraglia and Johns (2016). The model incorporates variables related to the person, the work, the organization, and the environment (i.e., the societal, economic, and cultural context) to classify antecedents and consequences of presenteeism. Although the model was developed on the basis of studies primarily considering presenteeism as an undesirable phenomenon, for this study, it was used to derive a system to categorize (potential) positive effects. Each of the four main categories comprises a different number of subcategories (see Table 1).

**Table 1:** Ratings of positive effects of presenteeism.

		***P*** **+** ***(*****N*****=136)***		***P – (*****N*****=30)***			***P*** **+** ***ratings after elimination of outliers (in %)***				
**Item content (subcategory)**	**Category**	***Mean***	***SD***	***Mean***	***SD***	***t*-value**	**Not agree at all (1)**	**Mainly not agree (2)**	**Agree partly (3)**	**Agree mainly (4)**	**Agree totally (5)**
Loyalty to professional standard	Individual	3.08	1.41	1.47	0.73	8.97^***^	19.1	16.9	21.3	22.1	20.6
Be a good example for colleagues	Work	2.97	1.19	1.60	0.93	5.79^***^	12.5	25.0	25.0	27.9	9.6
Impression management toward supervisor	Organization	3.32	1.29	1.97	1.22	5.23^***^	11.8	15.4	23.5	27.9	21.3
Do not want to let the sickness get me down	Individual	3.26	1.17	2.00	1.26	5.24^***^	6.8	15.2	32.6	29.5	15.9
No coverage needed	Work	3.89	1.35	2.70	1.39	4.33^***^	11.0	5.1	15.4	20.6	47.8
Demonstrate capacity to myself	Individual	3.06	1.28	1.90	1.30	4.48^***^	16.2	16.9	25.0	28.7	13.2
Handle workload	Work	3.95	1.22	2.80	1.45	4.04^***^	7.0	2.3	8.5	40.3	41.9
Be loyal to teammates	Work	3.38	1.26	2.27	1.31	4.33^***^	10.6	9.1	24.2	37.1	18.9
Contribute to social welfare of the society	Environment	2.83	1.21	1.77	0.97	4.51^***^	17.6	19.9	33.8	19.1	9.6
Avoid extra work for teammates	Work	3.98	1.19	3.00	1.39	3.95^***^	4.6	5.4	10.0	36.2	43.8
Adhere to social norms of the organization	Organization	2.78	1.19	1.83	1.23	3.91^***^	18.4	22.1	29.4	23.5	6.6
Expect a good performance review	Organization	2.79	1.35	1.90	1.27	3.30^**^	24.3	19.9	19.1	26.5	10.3
Being liked/accepted by teammates	Work	2.56	1.25	1.80	1.10	3.07^**^	27.9	20.6	24.3	22.1	5.1
Meet deadlines	Work	3.74	1.26	3.10	1.30	2.49^*^	8.8	8.8	16.2	32.4	33.8
Contribute to achievement of organization's goals	Organization	2.69	1.40	2.13	1.31	2.00^*^	28.7	19.1	18.4	22.1	11.8
Do relevant things on the way to/from work	Individual	2.13	1.26	1.57	0.90	2.88^**^	44.1	21.3	17.6	11.0	5.9
Maintain good team climate	Work	2.88	1.31	2.37	1.47	1.91+	20.6	18.4	25.0	24.3	11.8
Gain/maintain income	Individual	2.11	1.50	1.60	1.10	2.14^*^	55.1	15.4	8.1	5.9	15.4
Not have to forego private activities	Individual	2.48	1.31	2.07	1.17	1.59	32.4	20.6	20.6	19.9	6.6
Be able to socialize	Work	2.44	1.15	2.03	1.35	1.70+	26.5	26.5	27.2	16.2	3.7
Recover from health impairments	Individual	1.99	0.93	1.60	1.13	−0.96+	36.8	33.8	25.0	2.9	1.5
Contribute to economic growth	Environment	1.97	1.05	1.60	0.72	1.83+	43.4	27.9	18.4	8.8	1.5
Maintain career prospects	Organization	2.57	1.34	2.30	1.32	1.02	31.6	15.4	25.7	18.4	8.8
Not burden social security system	Environment	1.82	1.02	1.57	0.77	1.29	52.3	29.2	14.6	3.8	0.0

*P+: participants who had shown presenteeism during the past 12 months; P–: participants who had been ill during the past 12 months, but had not shown presenteeism. All items were rated on a 5-point scale, ranging from 1 = not agree at all to 5 = totally agree*.

+ *p < 0.10*,

* *p < 0.05*,

** *p < 0.01*,

*** *p < 0.001*.

The applicability of the model was previously tested in a qualitative online-survey with students working part-time. Participants were asked to describe potential positive effects of working while ill (Röser et al., 2021). The answers were content-analyzed and results revealed that three of four categories and their subcategories were required except for effects on the environment, which no one mentioned. This fact was attributed to the research design with open questions stimulating the recall of obvious effects rather than less apparent implications on the environment, such as consequences for the social welfare systems.

### Aims of the Current Study

The current study pursued two main targets. First, it was conducted in order to confirm the applicability of the content model (Lohaus and Habermann, 2019) for the description of potential and experienced positive effects in a sample of working adults. Thus, the following hypothesis was tested:

Hypothesis 1: The content model would be valid for positive effects of presenteeism experienced by working adults, i.e., categories and subcategories derived from the model are used to rate effects of presenteeism.

Second, the study aimed at the differences in the perception of positive effects of presenteeism between participants who had shown presenteeism and those who had not. Employees who view positive effects in attending work while ill are more likely to show presenteeism than employees who perceive positive effects to a lower degree. In accord with that assumption, we hypothesized the following:

Hypothesis 2: Employees who had shown presenteeism would rate the positive effects higher than employees who had not.

According to the health belief model (Janz and Becker, 1984), the assessment of costs and benefits influence the behavior. In addition, as postulated by Karanika-Murray and Biron (2020), two kinds of presenteeism are associated with positive effects. Thus, we expected that presenteeism is associated with the experienced positive outcomes, which resulted in the third hypothesis:

Hypothesis 3: Presenteeism propensity would be associated with (a) distinct positive effects and (b) an overall rating of positive effects.

## Methods

### Procedure

The study design was a cross-sectional online survey. The link to the questionnaire was distributed via social networks. Prior to participation, respondents were informed about the content of the survey, that participation was voluntary, and they could cancel it any time without facing disadvantages. They were informed that no personalized data would be collected, that their data would be used for scientific purposes only and would be stored and analyzed anonymously. The survey was online from 21.07.2020 until 28.07.2020. Of 293 people starting the questionnaire, 194 completed it (66.2%). The average processing time was 13.5 min.

### Measurement Instruments

Questions covered the current job (sector of work, size of organization, job title, characteristics of the employment relationship) followed by two questions asking how often during the past 12 months participants had stayed home due to illness (absenteeism) and attended work in spite of illness (presenteeism, see e.g., Aronsson et al., 2000; Gerich, 2016). The main part were 24 items concerning positive effects of presenteeism. Referring to the category system developed in a previous study (Röser et al., 2021), the items each started with the following phrase “Working in spite of illness had the tangible advantage that …” for those who had shown presenteeism and with “Working in spite of illness would have the tangible advantage that…” for those who had not. The second part of the items were phrased nearly identically in both conditions, e.g., “… I avoid(ed) extra work for my teammates.” Each item was presented with a 5-point rating scale (1 = “do not agree at all” to 5 = “totally agree”). Then participants had the opportunity to mention further positive effects. The main part closed with a single overall rating of positive effects of attending work while ill using the above mentioned scale. The questionnaire ended with demographic items (age, gender, supervisory duties, seniority, income, face time with clients).

### Data Processing

First, participants reporting long-term sickness (i.e., ≥ 60 days, see e.g., Gerich, 2016; Lohaus and Röser, 2019) were eliminated from the sample (*N* = 13). Presenteeism propensity was calculated as follows: The number of health events was calculated as the sum of presenteeism and absenteeism frequencies. We computed presenteeism propensity as presenteeism frequency divided by the number of health events. Outliers of ratings of positive effects were determined using SPSS. Five items had outliers with the numbers ranging from four to seven. These ratings were eliminated from the data set in order to test the applicability of the content model. Among those participants who reported days of sickness and thus presumably had the choice between presenteeism and absenteeism (*N* = 166), differences in perception of participants who had shown presenteeism and those who had not were tested via *t*-Tests in case of homogenous variances and Welch-Tests in case of heterogeneous variances. An exploratory principal component analysis (PCA) was performed in order to reduce the number of variables (Costello and Osborne, 2005) and to improve the subjects-to-variable-ratio for the subsequent multiple linear regression. The Kaiser criterion was used to identify the number of factors and varimax rotation to facilitate interpretation of factors. Linear regressions were computed to test the prediction of presenteeism propensity from the seven factors identified in the PCA and subsequently from the overall ratings of positive effects.

### Sample

After the described data cleansing 181 participants remained (49.2% female, 35.9% in a supervisory position). Age of participants ranged from under 25 to over 65 years with the strongest category being 36–45 years (31.5%). The average work experience was 14.8 years with 8.1 years with the current employer. Participants came from different sectors, with the social and educational sector (21%) and information technology (12.7%) most strongly represented. Fifteen participants reported 0 days of sickness during the previous 12 months (8.3%).

## Results

### Sickness, Presenteeism Prevalence, and Presenteeism Propensity

Participants reported an average of 14.3 days of sickness during the previous year. Of these, they stayed home on 6.6 days and attended work on 7.7 days on average. Presenteeism prevalence was distinguished between the complete sample and the subsample of participants reporting days of sickness (Navarro et al., 2018). The prevalence rate of the complete sample was 75% and of those who had been sick and thus were able to show presenteeism in the first place was 82%. Presenteeism behavior operationalized as presenteeism propensity among those who had been sick during the previous year (Gerich, 2016) was 0.49, which means, in 49% of cases of sickness the participants decided to attend work.

### Test of Hypotheses

Table 1 presents the means and standard deviations for positive effects of presenteeism distinguishing between participants who were sick during the previous 12 months and had shown presenteeism (P+) and those who were sick but had not shown presenteeism (P-). In addition, it lists the *t*-values for differences in ratings between both groups, and frequencies of scale points for positive effects of presenteeism only for participants who had shown presenteeism (P+).

In order to test hypothesis 1, that the content model offers a valid category system to describe positive effects of presenteeism by those who had shown presenteeism (*N* = 136), outliers were removed as described above. Then the percentages of ratings for each scale point were inspected. A subcategory (item) should be valid if at least one person partly agreed that attending work while sick had that particular positive effect. As can be seen in Table 1 (last three columns), that was the case for all 24 items. Thus, results supported hypothesis 1.

Hypothesis 2 posited that participants who had shown presenteeism rate positive effects higher than participants who had not shown presenteeism. Results presented in Table 1 reveal that only two items did not differ significantly between the groups. In order to counteract a possible problem of multiple comparisons, a Bonferroni correction was done. After that, only 12 of the 24 comparisons were significant. These were the items with *t*-values above 3.3 (i.e., those marked with ^***^ in Table 1, column “t-value,” and the item “Expect a good performance review”). Further, overall ratings (one single item and the mean of 24 items) were tested. The one-item overall rating of positive effects was significantly higher [*t*_(164)_ = 7.46, *p* < 0.001; Cohen's *d* = 1.02] for participants who had shown presenteeism (*M* = 3.24, *SD* = 1.03) than for those who had not (*M*= 1.70, *SD* = 0.99). In addition, the mean of positive effects was significantly higher [*t*_(164)_ = 6.46, *p* < 0.001; Cohen's *d* = 0.63] for participants who had shown presenteeism (*M* = 2.86, *SD* = 0.61) than for those who had not (*M* = 2.04, *SD* = 0.72). Consistent with hypothesis 2, all comparisons between groups were significant, and effect sizes for overall assessments of positive effects were high (Cohen, 1988).

The third hypothesis posited that positive effects are associated with presenteeism behavior. A PCA was performed in order to reduce the number of variables. The Kaiser–Meyer–Olkin measure of sampling adequacy was 0.803, representing a relatively good factor analysis. Bartlett's test of Sphericity was significant (*p* < 0.001), indicating that correlations between items were sufficiently large for performing a PCA. Only factors with eigenvalues ≥1 were considered. A varimax rotation yielded a well-interpretable solution, and although there were cross-loadings, most items loaded highly on only one factor with a minimum loading of 0.5 (Table 2). The seven factors solution explained 66% of the variance. We interpreted the factors as representing (1) social norms reflecting the (assumed) expectations of others within the organization, (2) economic considerations, (3) team spirit, (4) endurance, (5) getting one's work done, (6) side benefits in the sense of personal advantages that do not directly relate to one's work, and (7) making one's living. To examine hypothesis 3, multiple linear regressions were performed for the subsample of participants who had shown presenteeism. First, a multiple linear regression of presenteeism propensity on the seven factors of positive effects was calculated. Results are presented in Table 3. The seven factors of positive effects explained 16.4% of the variance in the criterion [*F*_(7, 128)_ = 4.77, *p* < 0.001], which represents a moderate effect (Cohen, 1988). In addition, a multiple linear regression was performed with the overall ratings of positive effects (one single item) and the mean of the positive effects (calculated from the 24 items on positive effects). Results can be found in Table 4. The positive effects (single-item rating and mean of 24 items) explained 12% of the variance in presenteeism behavior [*F*_(2, 133)_ = 9.76, *p* < 0.001], which represents a moderate effect (Cohen, 1988). These results are consistent with hypothesis 3.

**Table 2:** PCA rotated component matrix.

**Factor**	**Social norm**	**Economic** **considerations**	**Team spirit**	**Endurance**	**Do one's work**	**Side benefits**	**Make a living**
**Item (subcategory)**	**1**	**2**	**3**	**4**	**5**	**6**	**7**
Expect a good performance review	0.819	0.003	−0.073	−0.019	0.106	0.143	0.079
Impression management toward supervisor	0.809	−0.166	0.147	0.142	0.077	0.054	0.162
Be loyal to teammates	0.685	0.084	0.440	0.102	−0.135	0.106	−0.024
Maintain career prospects	0.681	0.075	−0.044	−0.112	0.233	0.236	0.167
Be a good example for colleagues	0.633	0.229	0.251	0.086	0.068	−0.175	0.052
Adhere to social norms of the organization	0.602	0.236	0.280	−0.063	−0.042	0.217	0.037
Being liked/accepted by teammates	0.575	0.265	0.345	0.090	−0.225	0.172	−0.222
Maintain good team climate	0.553	0.344	0.304	0.143	−0.142	0.250	−0.141
Loyalty to professional standard	0.501	0.121	0.267	0.347	0.001	−0.165	0.174
Contribute to economic growth	0.024	0.833	0.016	0.163	0.014	0.047	0.076
Not burden social security system	0.135	0.792	0.053	0.094	0.052	0.138	−0.071
Contribute to achievement of organization's goals	0.186	0.652	0.121	0.046	0.368	−0.001	0.370
Avoid extra work for teammates	0.204	0.060	0.770	0.018	0.065	0.047	−0.236
No coverage needed	0.224	−0.030	0.750	−0.152	0.061	0.040	0.240
Do not want to let the sickness get me down	0.214	0.163	−0.103	0.746	−0.004	0.080	0.003
Recover from health impairments	−0.307	0.000	0.036	0.661	0.008	0.222	−0.003
Demonstrate capacity to myself	0.421	0.211	−0.201	0.519	0.035	0.210	−0.268
Contribute to social welfare of the society	0.176	0.191	0.455	0.500	−0.142	−0.094	0.083
Meet deadlines	0.085	0.070	−0.112	−0.105	0.830	0.004	0.089
Handle workload	0.006	0.090	0.143	0.080	0.812	0.145	−0.139
Not have to forego private activities	0.139	−0.081	0.131	0.105	0.133	0.738	0.023
Do relevant things on the way to/from work	0.140	0.280	−0.201	0.124	0.075	0.644	−0.048
Be able to socialize	0.155	0.259	0.381	0.103	−0.116	0.555	0.297
Gain/maintain income	0.177	0.097	−0.018	−0.010	−0.035	0.071	0.868

*Gray formatted cells denote the allocation of items to factors*.

**Table 3:** Multiple linear regression of presenteeism propensity on the factors of positive effects (P+, *N* = 136).

**Item content**	**β**	***t***	***P***
Economic orientation	0.305	3.297	0.001^**^
Financial advantages	0.222	2.681	0.008^**^
Endurance	0.232	2.458	0.015^*^
Side benefits	−0.167	−1.886	0.062
Social norm	−0.173	−1.666	0.098
Team spirit	0.062	0.706	0.481
Do one's work	0.014	0.173	0.863
Adjusted *R*^2^	0.164^**^		

* *p < 0.05*,

** *p < 0.01*.

**Table 4:** Multiple linear regression of presenteeism propensity on overall positive effects.

**Item content**	**β**	***t***	***p***
Mean of positive effects	0.137	1.662	0.099
Overall rating of positive effects	0.308	3.740	0.000^***^
Adjusted *R*^2^	0.12^***^		

*(P+, N = 136)*.

*** *p < 0.001*.

## Discussion

The study had the objective of investigating positive effects of presenteeism among a sample of working adults. Results supported the three hypotheses. First, the content model of presenteeism is applicable to positive effects. Second, participants who showed presenteeism rated positive effects of the behavior higher than those who had not. Third, experienced positive effects related to presenteeism propensity. The results are discussed below.

First, participants perceived positive effects of presenteeism that relate to themselves, their work, the organization, and the environment. These results support the assumption that variables, which were identified as antecedents of presenteeism and related to negative effects of it (Miraglia and Johns, 2016; Lohaus and Habermann, 2019), are also relevant with regard to positive effects of presenteeism. Thus, the content model offers a common framework to investigate variables associated with positive and negative aspects of presenteeism.

Second, the fact that the majority of participants (82%) decided for presenteeism in case of illness is consistent with the health belief model (Janz and Becker, 1984) assuming that they perceive themselves as robust enough to work in spite of health impairments. The model presumes that employees will show presenteeism if the expected benefits outweigh the costs. The finding that participants that worked while ill perceived a higher degree of positive effects than those who did not show presenteeism is in accord with this presumption. Further, even employees who perceive benefits from presenteeism to be a bit lower than the respective costs might decide for presenteeism if there are work adjustments that allow them to work in spite of health impairments (Thun et al., 2013; Thun, 2017).

Third, the regression of presenteeism propensity on the seven factors of positive effects revealed significant associations for three of them. When participants decide for presenteeism, they obviously consider economic implications for their organization, the society, or themselves. Further, the “endurance”-factor predicted presenteeism, i.e., participants perceive that their health benefits from working. This finding corresponds to the predictions of the health belief model and to the conception of functional presenteeism as postulated by Karanika-Murray and Biron (2020). However, we did not find a convincing indication for therapeutic presenteeism since related aspects (represented in the “social norms”- and the “team spirit”-factor) were not significant. The regression of presenteeism propensity on the overall effects rendered the interesting result that the overall rating of positive effects was significantly related to presenteeism, while the mean of the 24 variables was not. It suggests that participants did not simultaneously take into consideration all 24 variables when making their overall assessment of positive consequences of presenteeism. It seems plausible to suppose that they either extended their evaluation to variables that were not included in the list or weighted them differently. The results suggest that employees who decide for presenteeism assume responsibility for the economic welfare of their organization and the society. This might be interpreted as an expression of employees' affective commitment, which is an established antecedent of presenteeism (Miraglia and Johns, 2016). Taking their strive to work in order to cope with their health impairments (represented in the “endurance”-factor) for granted, one could draw the conclusion that employees with less severe impairments should be supported to work. That could be achieved by work adjustments (Thun et al., 2013; Thun, 2017) and by assigning graded or partial absences in case of illness (Markussen et al., 2012; Godoy, 2016), which imply graded or partial presenteeism. The fact that positive effects concerning the team and the organization, such as team climate, staff level, and ease of replacement, did not relate significantly to presenteeism is interesting, because Miraglia and Johns (2016) identified them as relevant antecedents of presenteeism in their meta-analysis. The discrepancy might be explained by the employees' perception of these variables as representing rather attendance pressures (Saksvik, 1996) than experienced positive effects.

A strength of the study lies in its attempt to systematically investigate positive effects of presenteeism using an established model. However, some aspects limit the generalizability of the findings. The convenience sample gained via social media was relatively small and not representative of the population. That entails a restriction in the applicability of statistical methods and a small comparison group for participants who had not shown any presenteeism. Yet with regard to general presenteeism-related indicators, the results can be considered as valid: The sample comprises meaningful individuals with regard to work experience, health status during the previous year, and distribution of gender, age, and sector. Prevalence rates of presenteeism and presenteeism propensity were at levels comparable to other studies. Pohling et al. (2016) reported a prevalence rate of about 90% for a German working sample. Presenteeism propensity of a Canadian sample was 0.50 (Biron et al., 2006), and Gerich (2016) reported 0.59 for an Austrian sample of the working population. Thus, the results of the current study indicate comparability of samples with regard to presenteeism behavior. Overall, due to the small sample size and the cross-sectional design, results cannot be generalized.

The results of the study point to the importance of considering positive effects of presenteeism in a context of research that has hitherto mainly focused on its negative effects and on measures to reduce the behavior. It might be useful to differentiate circumstances in which presenteeism occurs and to take a closer look into how employees evaluate costs and benefits of presenteeism (Janz and Becker, 1984). With regard to the findings of researchers concerning the motives for presenteeism (e.g., Johansen et al., 2014), it appears worthwhile to investigate the relation between motives and positive effects. The importance of positive aspects of presenteeism with regard to practical implications cannot be overestimated. Should employees instead of showing presenteeism take recourse to absenteeism, the productivity loss and the resulting decline in personal incomes, company profits, and subsequently taxes and welfare subsidies (e.g., Goetzel et al., 2004) could lead to a lasting downturn. To counteract such a negative prospect at least in part, it is justified to adjust the work to less severe health impairments and reasonable to assign graded absences. The results of this study state positive effects that might result from these measures. However, although it is imperative to appreciate the positive effects of presenteeism as a stabilizing factor in the economies of today, caution is warranted to avoid an insidious long-term deterioration of the health of large parts of the workforce. This is even more important in view of the COVID-19 pandemic. It will be thrilling to see how societies cope with this challenge.

## Data Availability Statement

The raw data supporting the conclusions of this article will be made available by the authors, without undue reservation.

## Ethics Statement

Ethical review and approval was not required for the study on human participants in accordance with the local legislation and institutional requirements. The patients/participants provided their written informed consent to participate in this study.

## Author Contributions

DL designed the study and developed the questionnaire, analyzed the data, wrote the original manuscript, and revised the manuscript. WH provided data analysis ideas, checked the analyses, and revised manuscript. IEK developed the questionnaire and collected the data. FR checked the analyses and revised the manuscript. All authors contributed to the article and approved the submitted version.

## Conflict of Interest

WH is partner of H & L KarriereBeratung civil partnership in 64686 Lautertal, Germany. The remaining authors declare that the research was conducted in the absence of any commercial or financial relationships that could be construed as a potential conflict of interest.
